# SnakeCube: containerized and automated pipeline for de novo genome assembly in HPC environments

**DOI:** 10.1186/s13104-022-05978-5

**Published:** 2022-03-07

**Authors:** Nelina Angelova, Theodoros Danis, Jacques Lagnel, Costas S. Tsigenopoulos, Tereza Manousaki

**Affiliations:** 1 Hellenic Centre for Marine Research (HCMR), Former U.S. Base of Gournes, Institute of Marine Biology, Biotechnology and Aquaculture (IMBBC), P.O. Box 2214, 71003 Heraklion, Crete, Greece; 2 School of Medicine, University of Crete, 71003 Heraklion, Crete, Greece; 3 INRAE, UR1052, Génétique et Amélioration des Fruits et Légumes (GAFL), 67 Allée des Chênes, Centre de Recherche PACA, Domaine Saint Maurice, CS60094, 84143 Montfavet, France

**Keywords:** Assembly, Container, Pipeline, Genome, *de-novo*

## Abstract

**Objective:**

The rapid progress in sequencing technology and related bioinformatics tools aims at disentangling diversity and conservation issues through genome analyses. The foremost challenges of the field involve coping with questions emerging from the swift development and application of new algorithms, as well as the establishment of standardized analysis approaches that promote transparency and transferability in research.

**Results:**

Here, we present SnakeCube, an automated and containerized whole de novo genome assembly pipeline that runs within isolated, secured environments and scales for use in High Performance Computing (HPC) domains. SnakeCube was optimized for its performance and tested for its effectiveness with various inputs, highlighting its safe and robust universal use in the field.

**Supplementary Information:**

The online version contains supplementary material available at 10.1186/s13104-022-05978-5.

## Introduction

Modern sequencing technologies now allow scientists to generate de novo genome sequences for non-model organisms. However, routinely implementing such bioinformatics analyses remains challenging for both human and non-human resources. The time needed for manually controlling and pairing computationally heavy and memory straining tasks highlight the need for automation, standardization and chaining of the involved steps. Most existing workflows refer to the containerization of a single tool, may not involve containerization at all and may not be automated, with each step running individually in a manual chaining manner [1, 2]. Following the needs of the community, we aimed at building a complete genomic pipeline in a containerized form for use in HPC domains, which performs all analyses from the level of raw data processing and up to producing a fully polished assembly. We present SnakeCube, a de novo genome assembly pipeline which can operate with both long MinION and short Illumina reads. The workflow is based on our previous work on *Lagocephalus sceleratus* genome [3]. Furthermore, it is benchmarked and optimized using two additional publicly available datasets on distantly related organisms, with different genome sizes and raw data amounts, to monitor the response of the software.

## Main text

### Methods

The pipeline was constructed using the Snakemake workflow manager [4]. Then, it was isolated in a Singularity container for autonomy and integrity [5]. Snakemake splits the workflow into clearly defined individual steps (rules), which are either logical pieces of the procedure (i.e. assembly step, polishing step) or parts with different dependencies (i.e. different python versions). For each rule, the Conda package manager [6] creates unique environments that do not interfere with the host system and has pre-installed all required packages and libraries. In such environments, the user has full control while not granted elevated privileges on the host system. This feature makes containers suitable for multi-user domains. The environments, the definition files and the scripts of the pipeline, are all packed in container images that run as a single job in any linux-kernel based system, as long as they have Singularity pre-installed. The users have control over their analysis through a set of hyper-parameters declared in an external configuration file of a*.yaml* format, a Jason like, human-readable data-serialization language. The file contains parameters and their corresponding values to control variables of the applications included in the workflow. All results are saved into the user’s workspace, along with a directed acyclic graph (DAG) of the steps and a summary file with the status of the output files for validation.

**Fig. 1:**
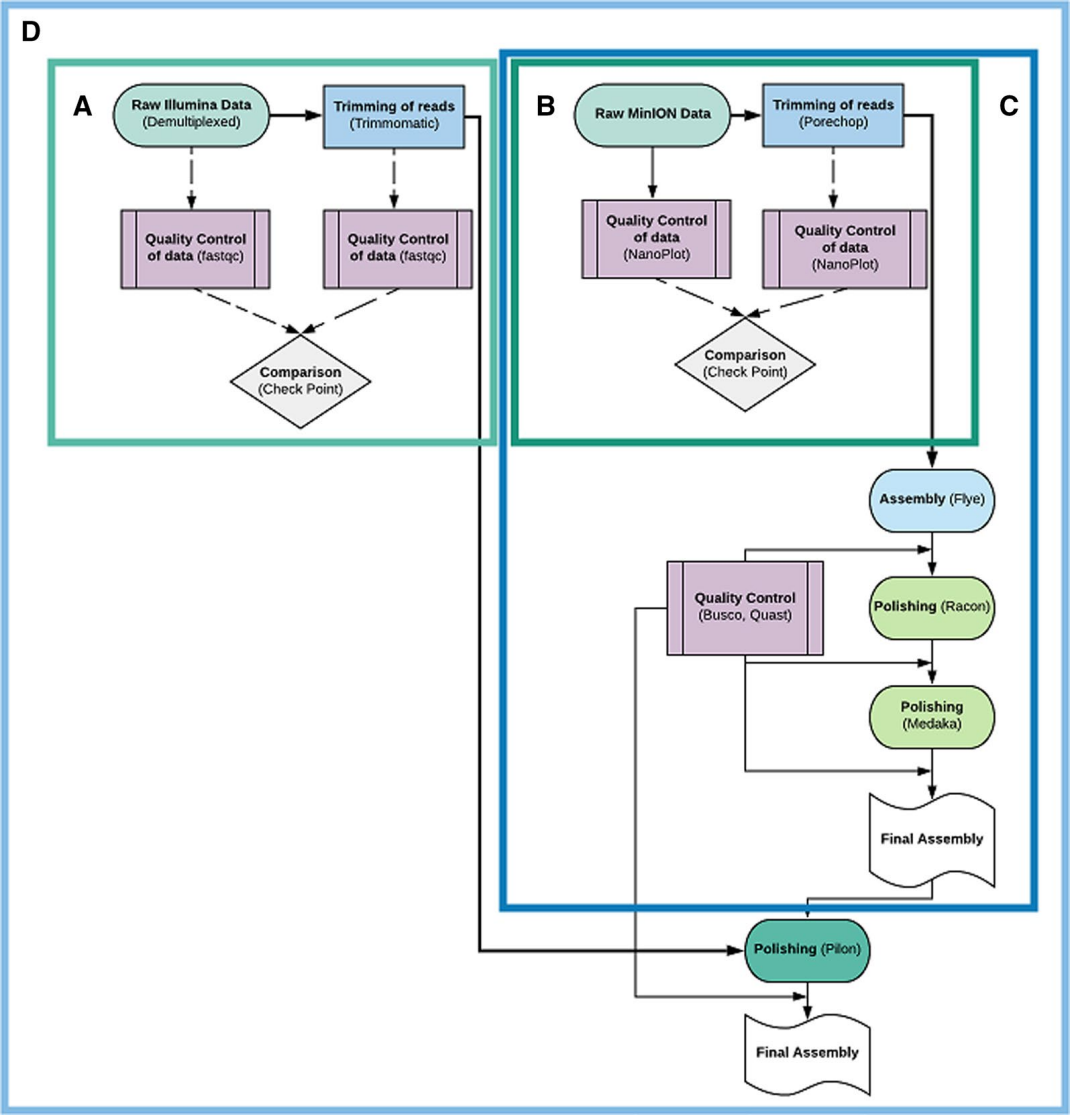
The workflow of SnakeCube and its sub-containers. Each box represents the different images available. **A** and **B** represent the quality checking steps for short or/and long reads. **B** and **C** serve users with only long reads. **D** combines them all, forming SnakeCube

The analysis itself is suited for building a de novo, fully polished, genome assembly for non-model species, with the use of both long (MinION, Oxford Nanopore Technology [ONT]) and short (Illumina) sequence reads (Fig. 1). The included steps are: 1) quality check and trimming of both short and long reads, 2) an initial genome assembly using the trimmed long reads, 3) polishing of the initial assembly using the long reads, and 4) final polishing of the genome with the trimmed short reads. The tools used in SnakeCube’s core, along with their description and versions, are presented in Additional file 1: Table 1. Some rules contain multiple tools and some tools are used in more than one rule. The analysis is also available in split sub-containers serving different needs and resources of the user.

**Table 1:** SnakeCube’s performance

Dataset	Raw-data size	Genome size estimation	Busco C, %	Published Busco, C%	Serial run time	Parallel execution time	Time Saved, %
*Hydropsyche tenuis*	8.3 Gb MinION, 18.8 Gb Illumina paired-end	219 Mb	98.2	98.3	39.04 h	35h 26m	10
*L. sceleratus*	9.68 GB MinION, 57,3 Gb Illumina paired-end	373 Mb	96.7	96.2	31.13 h	27h 29m	13
*Grus nigricollis*	116.5 Gb MinION, 54.6 Illumina paired-end	1.23 Gb	94.7	97.7	216.73 h	172h 47m	20.5

### Results

SnakeCube was first evaluated by reproducing the initial non-automated *L. sceleratus* analysis [3] used as the basis for the establishment of the pipeline. The produced output was generated as expected and SnakeCube scored similarly to the original work (Table 1). Then, the tool was benchmarked for its cpu load and memory usage while running serially, and it was optimized for speed and resource handling by parallel rule execution. The benefits of parallel execution and the general performance of SnakeCube were tested using two additional datasets of different organisms, to cover a range of genome sizes, raw data sizes and taxonomic groups: Caddisfly (insect) [7] and Crane (bird) [8].

#### Benchmarking and optimization

The benchmarking, optimization and testing of the pipeline took place in *zorba*, the HPC system of the Institute of Marine Biology, Biotechnology and Aquaculture (IMBBC, HCMR). *Zorba* is a high-performance computing cluster consisting of 328 cores and 2.3 TB memory at benchmarking time [21].

**Fig. 2:**
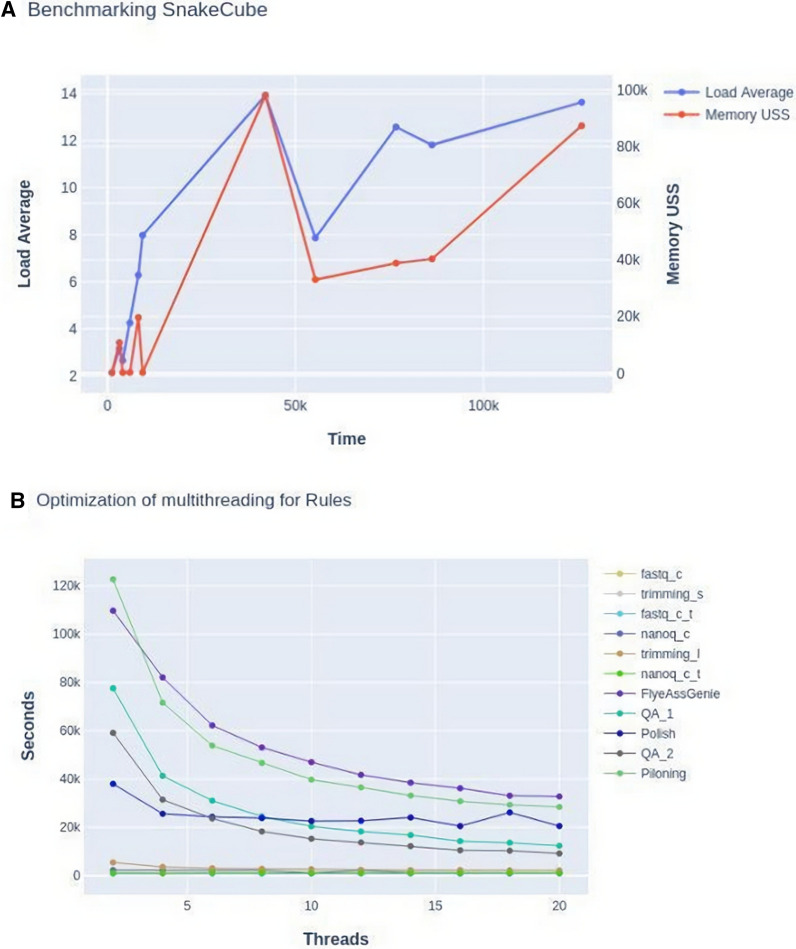
The benchmarking and optimization of SnakeCube based on the *L. sceleratus* dataset. **a** Reports of average memory and load monitoring records of three serial runs, with each point representing a rule of the container. **b** The rules were further independently monitored for their time-scaling efficiency when run multiple times with an increasing thread allowance. The memory properties are reported as in megabytes and only the highest value at any point is recorded. Time is measured in seconds. Rules are presented in the down-right side with their order of appearance

The cpu load recording was performed with the use of the Linux *watch* command, which was used to run all relevant commands at regular intervals, alongside *loadavg*, which gives the number of jobs in the run queue or waiting for input/output (IO), averaged in 15 m intervals. We monitored the whole pipeline in three repeated runs, and reported the average (Fig. 2a).

The memory straining of each individual task was monitored using the *benchmark* directive of Snakemake, and specifically the *max_uss* (Unique Set Size) field, which records the actual RAM used by the process without including the shared memory, and thus highlights the true cost of each step. Both cpu load and memory usage analysis did not show any sign of overloading. Even during the execution of demanding rules, the system reported idle cpus, and the memory usage was always lower than the given limit (128 GB RAM).

Subsequent parallelization was then introduced by dynamically determining the minimum number of threads required for each rule to reach its maximum computing-scaling efficiency. To identify the optimum cpu number per rule, we run the analysis multiple times incrementing the number of cpus and selected the point with no further speed up in the execution of each rule (Time vs Threads curve flattens). Based on this benchmarking, we specified the cpu threshold of each rule as a fraction of the overall host system cores, provided as a hyperparameter in the config file and via the *threads* directive of Snakemake (e.g. threads: int(config[“Available_Cores”]) * 0.5). Each rule was run using 1 to 10 pairs of cpus (10 runs) (Fig. 2b). Thus, the cpus that remained available during certain steps were used to run other independent rules in parallel. Non-demanding rules (e.g. summarizing software) were hardcoded to run in a single thread. On the contrary, workload intensive rules, like the main assembly construction, are always executed with full resources. Taking all benchmarks into account, the final optimization was formed using the *group* directive of Snakemake. Rules that did not directly depend on one another and could share proportions of the overall available resources, were grouped together for parallel execution. This resulted in three different execution groups. According to benchmarking results, the overall run time for each rule of the parallel pipeline was to some extent larger than the one observed in standalone runs, probably due to parallel jobs sharing the same resources (i.e. IO wait-time). However, the overall pipeline runtime was 10% to 20% reduced compared to the serial workflow execution depending on the dataset (Table 1). Overall, the parallelization seemed to be more effective in larger datasets.

#### Performance estimation

After benchmarking and optimizing SnakeCube, the final performance evaluation was based on the assembly completeness [9] and time efficiency for the three datasets (Table 1). In all datasets the parameters used were identical, except for the genome size (see Table 1) and the BUSCO taxonomy lineages used which were set to *actinopterygii*, *aves* and *insecta* for *L. sceleratus*, crane and caddisfly respectively.

### Conclusion

At its core, SnakeCube was designed to meet the needs of non-expert users or anyone who wishes to automate the genome assembly step of a project, saving time, effort and resources for subsequent analyses. The benchmarks and optimization of the software achieved good standardization and highlighted its robustness. We demonstrate SnakeCube is capable of accomplishing equally optimal results as those obtained in the original non-automated analyses used for testing the pipeline, as well as to perform efficiently for a range of inputs. Thus, it is a reliable new genomics software that can promote transparency and reproducibility in genome assembly projects of various fields, in times when genomes are massively produced and used in state-of-the-art research around the world. The general performance of the software varies, as expected, for different types of datasets and organisms, but is notably high, making SnakeCube a competitive, universal candidate for use in future research.

## Limitations

SnakeCube is restricted to run only in linux-based domains. Moreover, its efficiency highly depends on the resources of the host system and it is proportional to them. When it comes to the applications involved, SnakeCube uses a combination of specific bioinformatics tools, and although it reflects very good results for various datasets and organisms, may not work for all cases and users, due to the limitations inherited from the underlying software. Thus, although we tried to make the workflow as universal as possible, we acknowledge that this may not always be feasible.

## Supplementary Information

**Additional file 1:** **Table S1.** Tools and rules used by SnakeCube.

## Data Availability

SnakeCube is Python and bash based, and available via an open source license at GitHub: https://github.com/genomenerds/SnakeCube. The final deliverables (containers) work in any Linux-based system with Singularity pre-installed. The Crane, Caddisfly and *Lagocephalus sceleratus* datasets analysed during this study are included in their corresponding published articles [and their supplementary information files]. All benchmarking code is available on the GitHub repository of SnakeCube.

## Abbreviations

HPC: High Performance Computing
DAG: Directed acyclic graph
ONT: Oxford Nanopore Technology
