# Supplementary material for: SnakeCube: containerized and automated pipeline for de novo genome assembly in HPC environments
Source: BMC Res Notes. 2022 Mar 7;15:98. doi: 10.1186/s13104-022-05978-5 (PMC8900408; doi:10.1186/s13104-022-05978-5)
Supplement: Supplementary file 1 — Additional file 1: Table S1. Tools and rules used by SnakeCube. [file 13104_2022_5978_MOESM1_ESM.docx]

**Table S1:** Tools and rules used by SnakeCube.

| **Tools** | **Description** | **Version** | **Rule Participation** | **Rule Description** |
| --- | --- | --- | --- | --- |
| fastqc [10] | Quality (short reads) | 0.11.8 | fastq_c, fastq_c_t | Quality assessment of short reads (raw, trimmed) |
| Trimmomatic [11] | Trimming (short reads) | 0.39 | trimming_s | Trimming short reads |
| Multiqc [12] | Quality summaries | 1.6 | multiq_c, multiq_c_t | Summary of fastqc reports |
| NanoPlot [13] | Quality (long reads) | 1.29.0 | nanoq_c, nanoq_c_t | Quality assessment of long reads (raw,trimmed) |
| Porechop [14] | Trimming (long reads) | 0.2.3 | trimming_l | Trimming long reads |
| Flye [15] | Assembler | 2.6 (Internal: KmerGenie: v1.70.16) [16] | FlyeAssGenie | Genome Assembly |
| Quast [17] | Quality checking of assembly | 5.0.2 | QA_1, QA_2 | Quality assessment of the assembled genome |
| Busco [9] |  | 3.0 (Internal Blast: v2.2) |  |  |
| Racon [18] | Polishing (long reads) | 1.4.12 | Polish | Polishing with long reads |
| Medaka [19] |  | 0.9.2 |  |  |
| Pilon [20] | Polishing (short reads) | 1.23 | Piloning | Polishing with short reads |
